# MicroRNA 17-92 Cluster Mediates ETS1 and ETS2-Dependent RAS-Oncogenic Transformation

**DOI:** 10.1371/journal.pone.0100693

**Published:** 2014-06-26

**Authors:** Mohamed Kabbout, Duaa Dakhlallah, Sudarshana Sharma, Agnieszka Bronisz, Ruchika Srinivasan, Melissa Piper, Clay B. Marsh, Michael C. Ostrowski

**Affiliations:** 1 Department of Molecular and Cellular Biochemistry, College of Medicine, The Ohio State University, Columbus, Ohio, United States of America; 2 Graduate Program in Molecular Cellular and Developmental Biology, The Ohio State University, Columbus, Ohio, United States of America; 3 Solid Tumor Program, Comprehensive Cancer Center, The Ohio State University, Columbus, Ohio, United States of America; 4 Dorothy M. Davis Heart and Lung Research Institute, The Ohio State University, Columbus, Ohio, United States of America; 5 Division of Pulmonary, Allergy, Critical Care, and Sleep Medicine, Department of Internal Medicine, College of Medicine, The Ohio State University, Columbus, Ohio, United States of America; H.Lee Moffitt Cancer Center & Research Institute, United States of America

## Abstract

The ETS-family transcription factors *Ets1* and *Ets2* are evolutionarily conserved effectors of the RAS/ERK signaling pathway, but their function in *Ras* cellular transformation and biology remains unclear. Taking advantage of *Ets1* and *Ets2* mouse models to generate *Ets1/Ets2* double knockout mouse embryonic fibroblasts, we demonstrate that deletion of both *Ets1* and *Ets2* was necessary to inhibit *Hras^G12V^* induced transformation both *in vitro* and *in vivo. Hras^G12V^* expression in mouse embryonic fibroblasts increased ETS1 and ETS2 expression and binding to cis-regulatory elements on the *c-Myc* proximal promoter, and consequently induced a robust increase in MYC expression. The expression of the oncogenic microRNA 17-92 cluster was increased in *Hras^G12V^* transformed cells, but was significantly reduced when ETS1 and ETS2 were absent. MYC and ETS1 or ETS2 collaborated to increase expression of the oncogenic microRNA 17-92 cluster in *Hras^G12V^* transformed cells. Enforced expression of exogenous MYC or microRNA 17-92 rescued *Hras^G12V^* transformation in *Ets1/Ets2*-null cells, revealing a direct function for MYC and microRNA 17-92 in ETS1/ETS2-dependent *Hras^G12V^* transformation.

## Introduction

Cancer cells exhibit unique transformation properties that include independence from mitogenic and growth signals, unresponsiveness to anti-growth signals, escape from apoptosis and senescence, changes in gene expression, and acquired invasion and metastastatic capabilities [1]. *RAS* gene family activating mutations are present in 30% of all human cancers, and cells harboring *RAS* mutations have self-sufficiency in growth signals [2]. RAS is a GTP-binding protein that activates cellular proliferation and survival among other biological functions in response to extracellular signals under normal conditions [3], [4]. Chemotherapeutic agents targeting mutated RAS as well as upstream and downstream regulators have thus far failed to have significant clinical benefits in the treatment of cancer patients [5], highlighting the need to define the downstream effectors of the *RAS* pathway.

ETS1 and ETS2 are members of the ETS family of transcription factors and are downstream effectors of the RAS/RAF/ERK pathway [6]–[9]. These factors regulate genes involved in cellular proliferation, differentiation, apoptosis and transformation [9]. Ets1 and Ets2 share two highly conserved domains: the DNA binding domain at the C-terminal end, and the RAS/ERK activated Pointed domain at the N-terminal end [6]–[9]. Overexpression of dominant-negative forms of several ETS factors, including ETS1 or ETS2 block *Ras* transformation [10], [11], suggesting that ETS family members play a crucial role in this process. However, specific deletions of ETS family members is a more accurate approach for understanding the function of individual family members in *Ras* transformation. For example specific deletion of *Ets2* alone failed to inhibit *Ras* transformation in ES-cell derived fibroblasts [12].

Given the high homology between ETS1 and ETS2 protein structures, we hypothesized that ETS1 could be compensating for a loss of ETS2 in driving *Ras*-mediated transformation. Thus, we generated *Ets1* and *Ets2* null alleles in mouse embryonic fibroblasts using the Lox/Cre technology. We show that specific deletion of *Ets1* and *Ets2* impaired the *Hras^G12V^* transformation. Gene expression analysis and chromatin immunoprecipitation (ChIP) revealed that *Myc* and its downstream target *miR-17-92* were transcriptionally activated by ETS1 and ETS2 in response to *Hras^G12V^* expression. Overexpression of MYC or microRNA 197-93 (miR-17-92) rescued the impaired *Hras^G12V^* transformation in *Ets1/Ets2* deleted cells. These findings demonstrate that *Ets1* and *Ets2* are essential mediators of *Hras^G12V^* transformation, and revealed an oncogenic function for miR-17-92 in mediating Ras/Ets1/Ets2 transformation.

## Results

### *Ets1* and *Ets2* double knockout ablates *Hras^G12V^* transformation of MEFs

**Figure 1:**
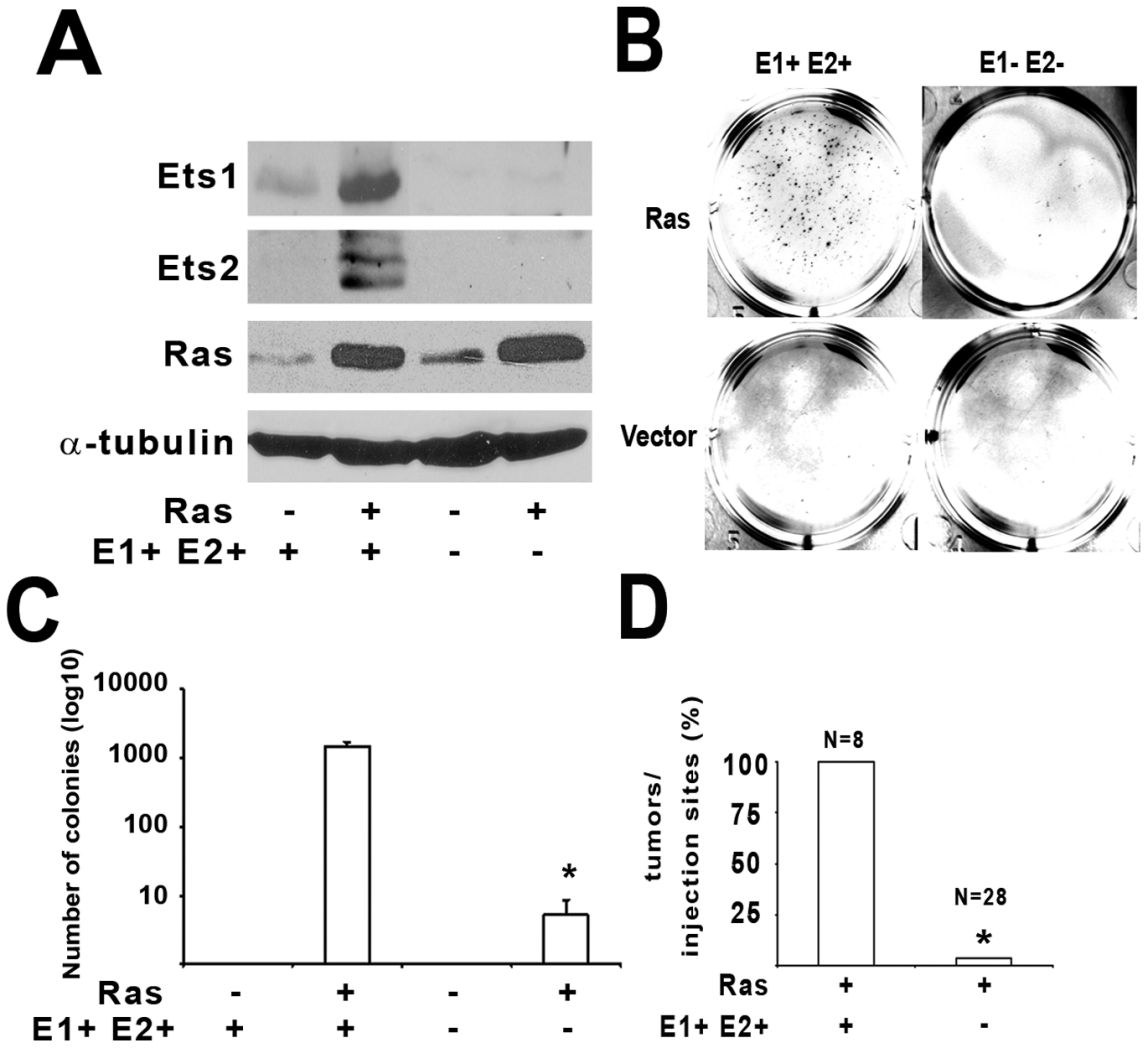
Deletion of both *Ets1* and *Ets2* ablates *Hras^G12V^* transformation of MEFs. *E1+E2+* and *E1−E2−* MEFs were infected with *HRasv^G12V^* or empty vector control retrovirus, then selected with Hygromycin for at least 5 days before functional examination of the four generated cellular genotypes, as shown. A) Western blotting analysis of 20 µg protein lysates probed with antibodies against proteins shown, with α-tubulin as loading control. B) Representative images and C) Quantification of the cellular colonies that grew in soft agar from the four different genetic groups Asterisk indicates P<0.05 as determined by the Student *t* test. D) Cells were injected subcutaneously into nude mice (10^6^ cells per injection site) and after 3 weeks the tumors were harvested. The ratios represent the percentage of tumors that grew from the total number of injections for each of the different genotypes.

ETS1 and ETS2 share high homology in their DNA binding and Pointed domains, and several studies have revealed that dominant-negative forms of these factors ablate *Ras* dependent transformation [7], [10], [11], [13]. We therefore hypothesized that both *Ets1* and *Ets2* might be required for efficient *Ras* transformation of MEFs. To test this hypothesis, we established spontaneously immortalized *Ets1^−/−^ Ets2^fl/fl^* MEFs from double transgenic mice, and stably expressed the Cre recombinase protein using a retroviral vector to generate double knockout *Ets1^−/−^ Ets2^−/−^* cells (*E1−E2−*). We observed efficient *Ets2* deletion in Cre-transduced cells as determined by detection of the *Ets2* null allele and absence of the *Ets2^flox^* allele (Figure S1A). To determine the effect of *Ras* transformation in the double-knockout MEFs, we infected both *E1+E2+* and *E1−E2−* cells with a *Hras^G12V^* retroviral vector containing a hygromycin marker and used drug selection to produce a stable mixed population of cells expressing *Hras^G12V^* and control cells tranduced with the hygromycin gene. Western blot analysis demonstrated that ETS1 and ETS2 expression is very low in the immortalized MEFs, but expression of both ETS1 and ETS2 increased dramatically in response to *Hras^G12V^* introduction (Figure 1A). In contrast, neither ETS1 nor ETS2 could be detected in double knockout *E1−E2−* MEFs in *Hras^G12V^* expressing cells (Figure 1A).

*E1−E2−* and *E1+E2+* cells had indistinguishable growth in monolayer cultures indicating that loss of the ETS-factors did not affect normal cell growth (Figure S1B). When grown in monolayer for 6 days, both *E1+E2+* and *E1−E2−* MEFs became contact inhibited, as demonstrated by flow cytometry analysis of propidium iodide labeled cells. Flow cytometry of these cell populations also revealed no significant differences in the sub-G0 peak, indicating no major differences in cell apoptosis between the double knockout and control cells (Figure S1C). As expected, *E1+E2+/Hras^G12V^* cells continued to grow after confluency, demonstrated by 3–4 fold higher number of cells in S-phase as determined by either BrdU labeling or flow cytometry (Figure S1D and S1E, respectively). In contrast *E1−E2−/Hras^G12V^* cells were contact-inhibited similar to the non-transformed MEFs (Figure S1D and S1E). Further, anchorage independent growth assays demonstrated that *E1− E2−/Hras^G12V^* MEFs formed only a few, small colonies in soft agar unlike *E1+ E2+/Hras^G12V^* MEFs, which formed numerous large colonies (Figure 1B, quantified in Figure 1C). MEFs that lacked either *Ets1*or *Ets2* alone formed approximately 2-fold fewer colonies in soft agar assays compared to cells with both genes intact, but formed 100-fold more colonies in the soft agar assay compared to the double-knockout cells (Figure S2A).

Consistent with the i*n vitro* analysis, a xenograft mouse model showed that subcutaneous injection of *E1− E2−/Hras^G12V^* cells developed one single tumor out of 28 injected sites, in contrast to *E1+ E2+*/*Hras^G12V^* cells, which formed 8 tumors out of 8 injected sites (Figure 1D and Figure S2B). Notably, genotyping of the single tumor that grew from *E1− E2−/Hras^G12V^* cells demonstrated that it contained the *Ets2^flox^* allele, and therefore still expressed ETS2. This finding further confirmed the requirement for both *Ets1* and *Ets2* for *Hras^G12V^* transformation (Figure S2C).

### *c-Myc* is required for *Ets1* and *Ets2*-mediated *Hras^G12V^* transformation

**Figure 2:**
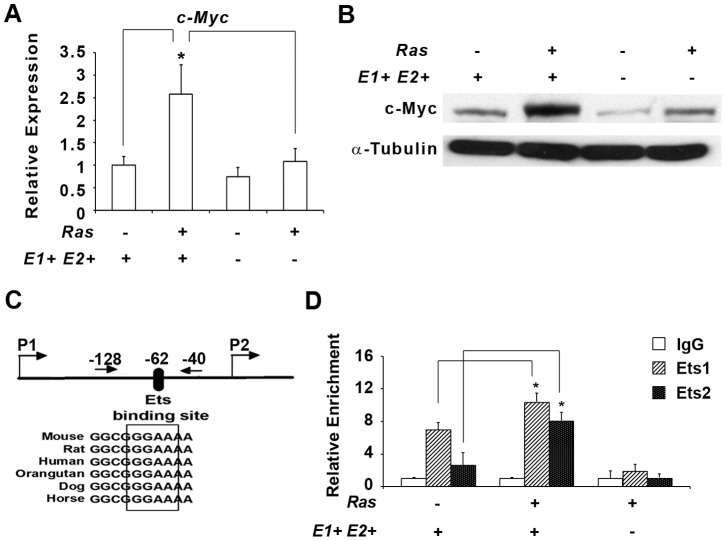
*Ets1* and *Ets2* activate *c-Myc* expression in *Hras^G12V^* transformed MEFs. A) Fold change of *c-Myc* gene expression by real time rt-PCR in the 4 different genetic groups examined. B) Western blotting analysis of 20 µg protein lysates probed with antibodies against MYC and α-tubulin. C) Schematic illustration of the *c-Myc* promoter showing conserved Ets binding sites (box) and the ChIP primers used (arrows) relative to the P2 promoter. D) ChIP performed on MEFs with indicated genotypes using anti-ETS1 and ETS2 antibodies with IgG as control. The threshold value for the promoter being studied was normalized to that of input values and represented as relative enrichment. Asterisk indicates P<0.05.

The *c-Myc* proto-oncogene was previously identified as a mediator of *Ras/Et*s transformation, but whether *c-Myc* is a direct target of ETS factors has not been conclusively established [11]. Consistent with previous results, analysis of *c-Myc* RNA expression in control and experimental genotypes revealed 2.5-fold increase in *E1+E2+/Hras^G12V^* cells compared to non-transformed controls and *E1−E2−/Hras^G12V^* cells (Figure 2A). Similarly, MYC protein was elevated in *E1+E2+/Hras^G12V^* cells compared to the other genotypes (Figure 2B). *In silico* analysis of the *c-Myc* proximal P2-promoter revealed a GGAA ETS*-*binding motif that is conserved in mammals (Figure 2C). Chromatin immunoprecipitation (ChIP) in *E1+E2+/Hras^G12V^* cells showed 2-fold enrichment of ETS1 and 4-fold enrichment of ETS2 on the *c-Myc* promoter relative to *E1+E2+* control cells, while ETS1 and ETS2 binding on the *c-Myc* promoter in *E1−E2−/Hras^G12V^* MEFs was no different than the IgG control (Figure 2D).

**Figure 3:**
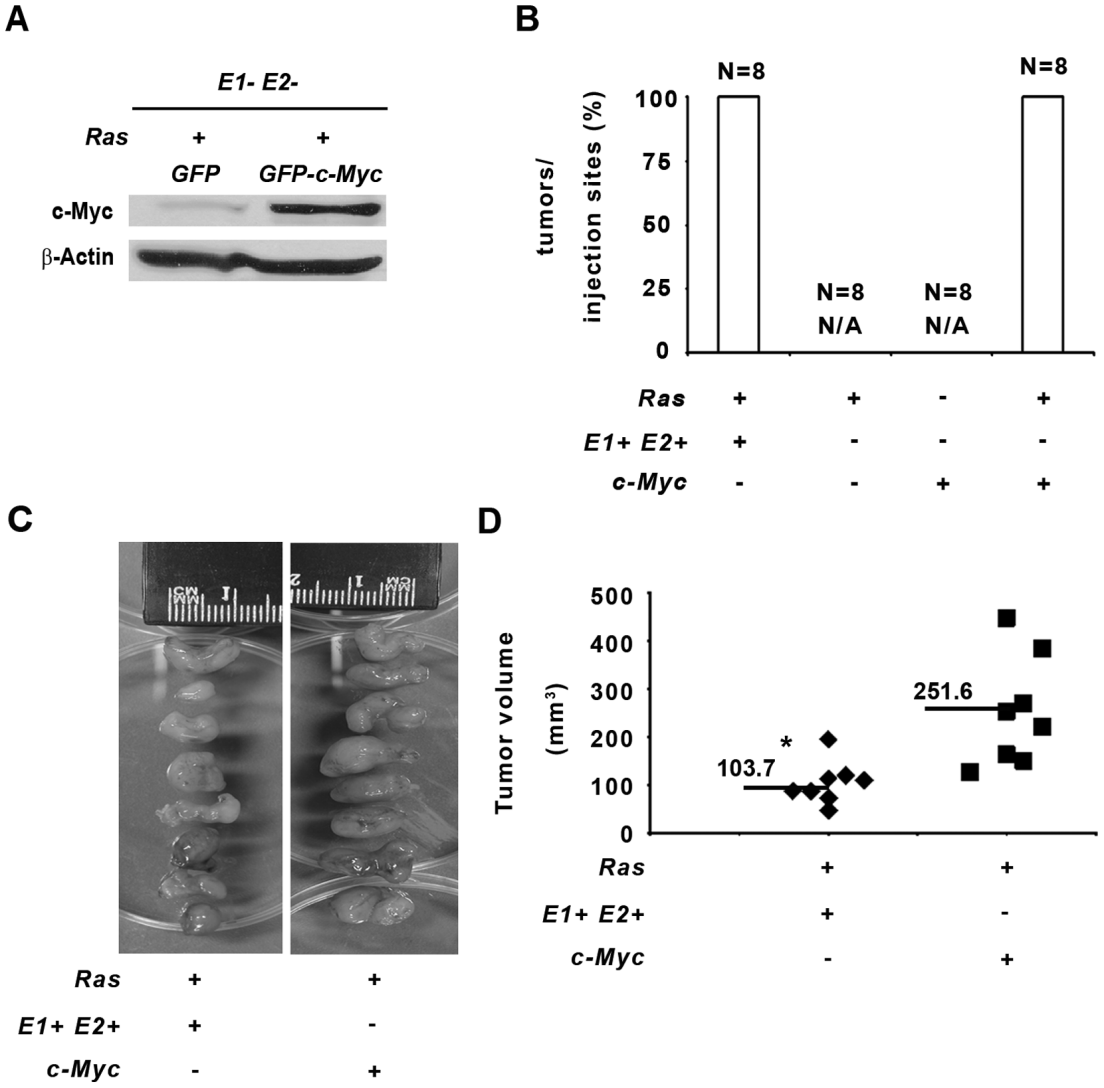
Overexpression of *c-Myc* in *Ets1/Ets2*-null MEFs rescued *Hras^G12V^* transformation. *E1−E2−* control and *E1−E2−/H-Rasv^G12V^* cells were infected with either MSCV-*GFP* empty control or MSCV-*GFP-c-Myc* vector, and cells were sorted for GFP expression by FACS. A) 20 µg of protein lysates from *E1−E2−/H-Rasv12/MSCV-GFP control* and *E1−E2−/H-Rasv12/MSCV-GFP-c-Myc* cells analyzed by western blot against MYC and β-Actin (protein loading control) antibodies. B) Graph representing the percentage of tumors formed over the total number of injections for the indicated cellular genotypes. N/A indicates that there were no tumors observed for the specified group. C) Representative pictures of all tumors derived from *E1+E2+*/*H-Rasv12* and *E1−E2−/H-Rasv12/MSCV-GFP-c-Myc* cells. D) Graph showing individual and average volumes of *E1+E2+*/*H-Rasv12* and *E1−E2−/H-Rasv12/MSCV-GFP-c-Myc* tumors. Asterisk indicates P<0.05.

In order to understand the biological significance of *Ets1* and *Ets2* transcriptional activation of *Myc* in *Hras^G12V^* transformation, we exogenously expressed *Myc* in *E1− E2−* and *E1−E2−/Hras^G12V^* MEFs using a *MSCV-GFP-Myc* retroviral vector, and sorted cells by FACS to produce a stable mixed population of GFP-MYC expressing cells (Figure 3A). When injected into nude mice, all 8 sites injected with *E1−E2−/Hras^G12V^*/*MSCV-GFP-c-Myc* MEFs developed tumors, while expression of MYC alone was not sufficient to transform *E1−E2−* MEFs (Figure 3B). The average tumor volume of *Myc*-rescued *E1−E2−/Hras^G12V^* was significantly larger than tumors from *E1+E2+/Hras^G12V^* cells (Figure 3C–D), likely because MYC was overexpressed in the rescued cells.

### The *miR-17-92* cluster is required for *Ets1/Ets2*-dependent *Hras^G12V^* transformation

MYC regulates different biological processes including microRNA expression. The *miR-17-92* cluster belongs to a network of MYC activated microRNAs [14], and its increased expression is associated with a variety of hematopoietic and solid tumor malignancies [15], [16]. Recent results showing that *miR-17-92* collaborates with activated *Hras^G12V^* and the adenovirus-encoded *E1A* oncogene to induce transformation in primary human fibroblasts further suggest this cluster as a potential candidate downstream of *c-Myc* and *Ets1/Ets2* [17].

**Figure 4:**
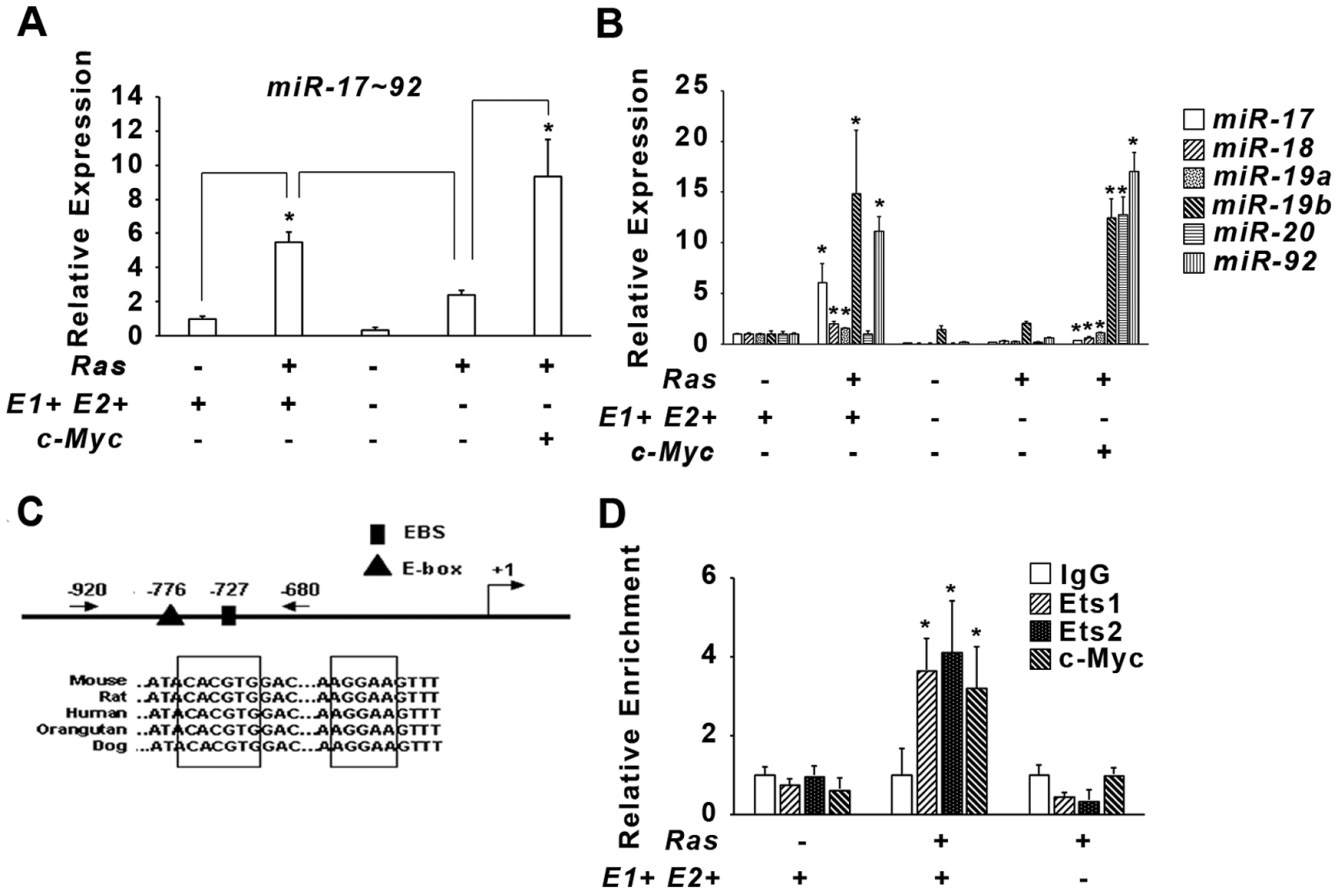
*mir17-92* expression depends on *Ets1* and *Ets2* in *Hras^G12V^* transformed MEFs. A) Fold change of pre-*mir17-92* and B) individual *microRNAs* of the *mir17-92* cluster: in the indicated genetic groups relative to control cells. Asterisk indicates P<0.05. C) Schematic illustration of the *mir17-92* promoter showing conserved ETS(box) and MYC (triangle) binding sites, as well as the ChIP primers used (arrows) relative to the start RNA start site. D) ChIP performed on the indicated MEFs genotypes using IgG control and specific antibodies as indicated. Asterisk indicates P<0.05.

Consistent with this hypothesis, precursor-*miR-17-92* (*pre*-*mir-17-92*) expression was increased approximately 5-fold in *E1+E2+ Hras^G12V^* transformed MEFs compared to control, while expression was decreased 3-fold in *E1−E2− Hras^G12V^* cells (Figure 4A). Exogenous expression of *c-Myc* in *E1−E2− Hras^G12V^* induced expression of *pre-mir-17-92* 4.5-fold compared to the double-knockout cells (Figure 4A). The *mir-17-92* cluster encodes six mature miRNAs, and expression analysis of all six mature miRNAs demonstrated that 5/6 were significantly upregulated in *E1+E2+/Hras^G12V^* MEFs compared to the wild-type control, while expression of all 6 was decreased in *E1−E2−/Hras^G12V^* compared either control or *E1+E2+/Hras^G12V^* MEFs (Figure 4B). Overexpression of *c-Myc* in *E1−E2− Hras^G12V^* cells resulted in rescue of expression for all 6 miRNAs (Figure 4B). However, the relative levels of the mature miRNAs differed in *E1+E2+ Hras^G12V^* cells compared to the *c-Myc*-rescued double-knockout cells, perhaps reflecting that high levels of MYC expression may alter miRNA processing [18].

**Figure 5:**
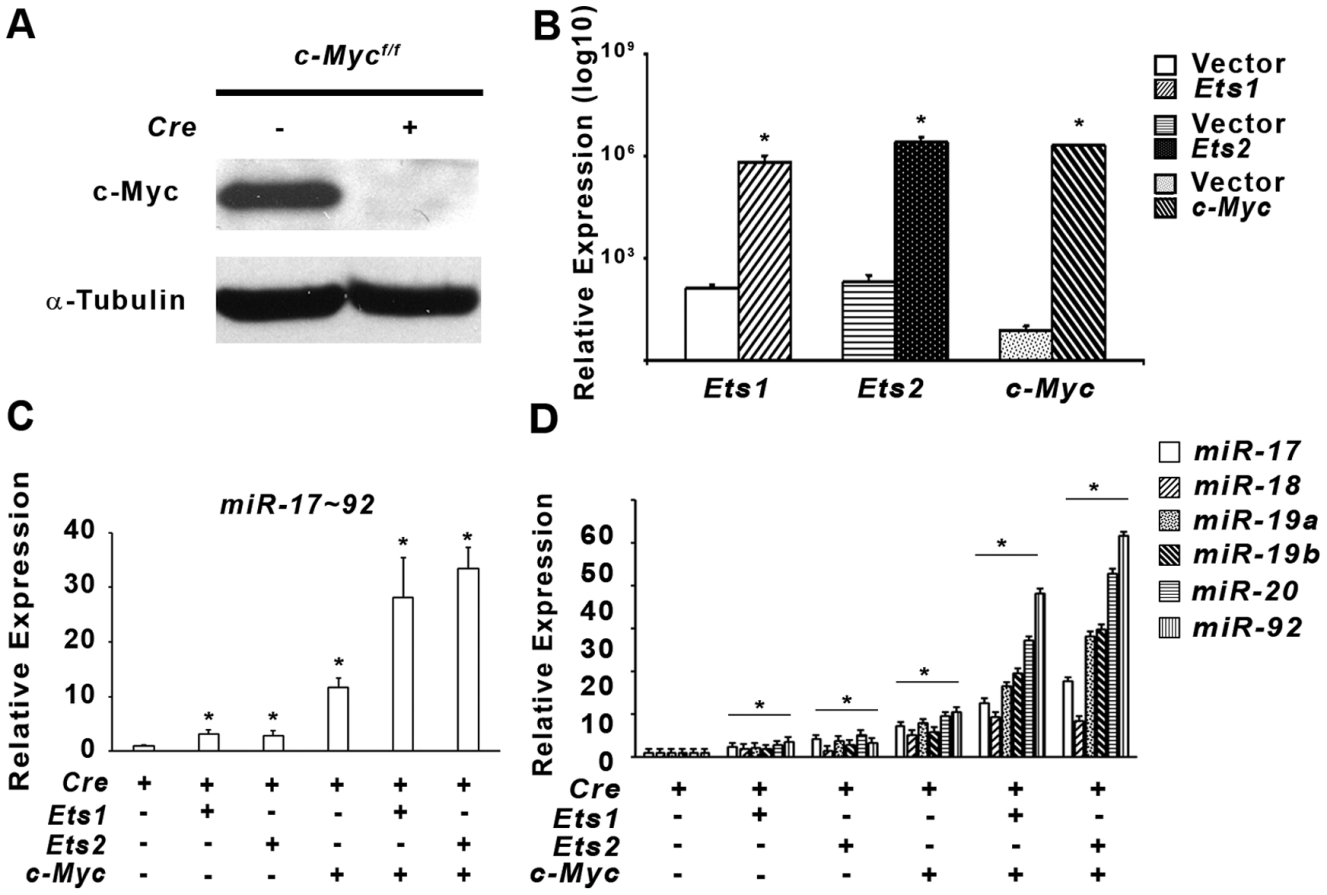
*Ets1*, *Ets2* and *c-Myc* activate miR17-92 transcription. *C-Myc^f/f^* MEFs were infected with *pBabe-Cre* retroviral vector or control *pBabe-empty* vector, selected with puromycin before functional examination. A) Protein analysis of 20 µg lysates from MEFs by western blot using antibodies as indicated B) Relative expression of *Ets1*, *Ets2* and *c-Myc* mRNAs after 48 hrs of transfection of expression vector. Asterisk represents P<0.05. C and D) Fold change relative real time PCR gene expression of pre-*mir17-92* (C) and individual miRs of the cluster (D) after 48 hrs transient transfection of the indicated vectors in *c-Myc^−/−^* MEFs relative to control empty vector. Asterisk indicates P<0.05.

The lower expression of both *pre-mir-17-92* and the mature miRNAs in *E1−E2−* cell*s* suggested that *Ets1/Ets2* could regulate the *miR-17-92* cluster independent of *c-Myc*. Analysis of the region 2 kilobase pairs upstream of *miR-17-92* cluster identified an ETS-binding motif conserved in mammals that is approximately 50 base pairs from a previously identified MYC binding site [14] (Figure 4C). Quantitative ChIP analysis revealed that ETS1, ETS2 and MYC binding is enriched in this proximal region in *E1+ E2+ Hras^G12V^* transformed MEFs compared to non-transformed wild-type and *E1− E2− Hras^G12V^* MEFs (Figure 4D). In order to test whether the binding of these factors was functional, we studied overexpression of the ETS factors in immortalized MEFs that lacked *c-Myc* (Figure 5A). Transient overexpression of *Ets1*, *Ets2* and *c-Myc* in *c-Myc^−/−^* cells was verified by qRT-PCR analysis (Figure 5B). Transient overexpression of *Ets1* or *Ets2* alone increased expression of *pre*-*mir-17-92* approximately 3-fold, while *c-Myc* alone increased the expression 11-fold compared to control (Figure 5C). Co-transient overexpression of *Myc* with either *Ets1* or *Ets2* resulted in a robust 30-fold superactivation of *miR-17-92* expression relative to control (Figure 5C). Similarly, the expression of all 6 mature miRNAs increased in response to overexpression of the three transcription factors (Figure 5D).

**Figure 6:**
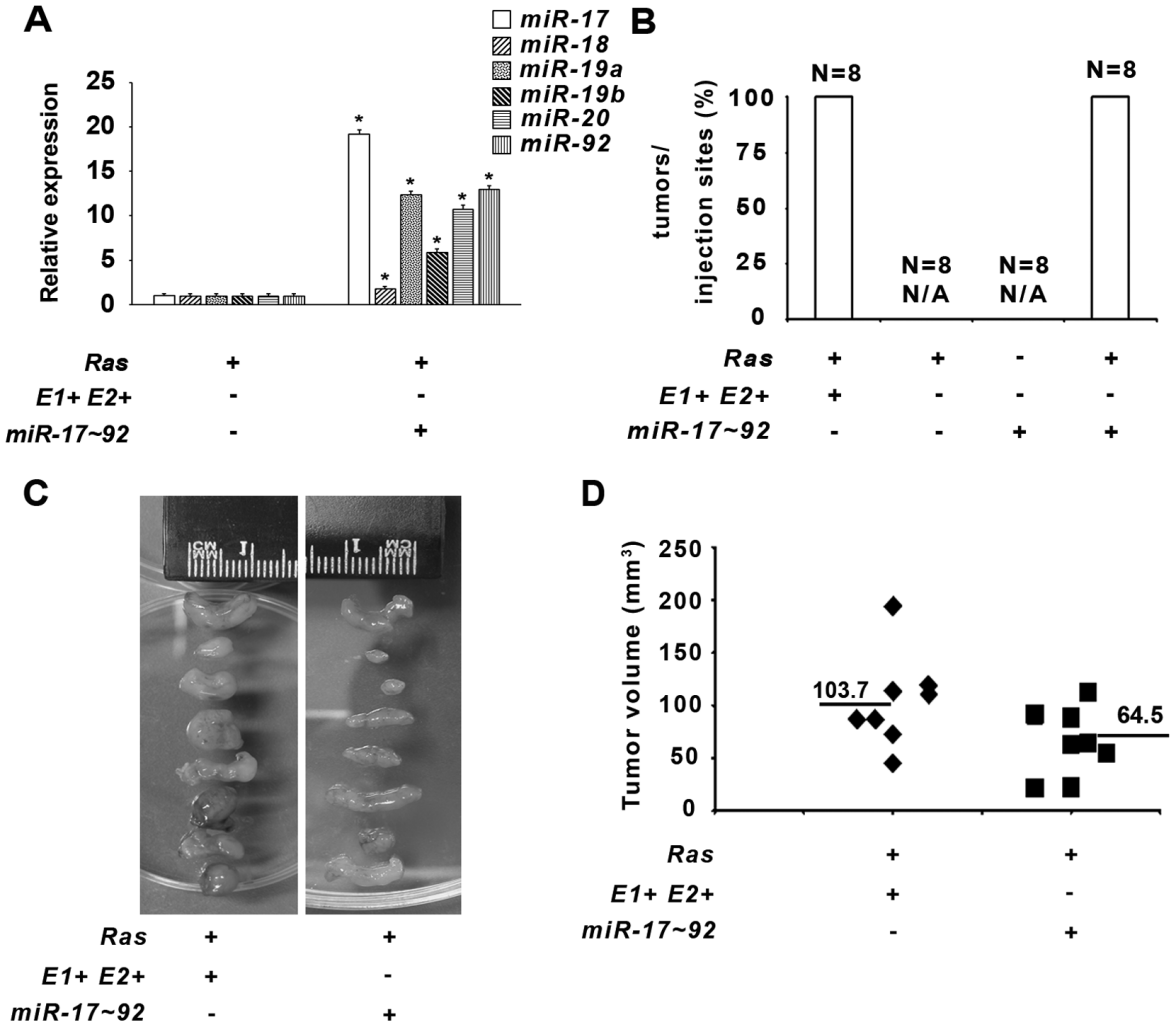
*MiR-17-92* overexpression in *Ets1/Ets2*-null MEFs rescue *Hras^G12V^* transformation. *E1−E2−/pBabe* control and *E1−E2−/H-Rasv12* were infected with either MSCV-puro empty control or MSCV-puro-*miR-17- 92* vector and cells were selected by Puromycin before further functional analysis. A) pre-*mir17-92* cluster expression in the indicated genotypes relative to control vector. Asterisk indicated P<0.05. B) Graph demonstrating the percentage of tumors formed over the total number of injections for the different cellular groups. N/A indicates that there were no tumors observed for the specified group. (C) Representative images showing the total *E1+E2+*/*H-Rasv12* and *E1−E2−/H-Rasv12/MSCV-puro-miR-17-92* derived tumors. D) Graph indicating individual and average volume of *E1+E2+*/*H-Rasv12* and *E1−E2−/H-Rasv12/MSCV-puro-miR-17-92* tumors.

To assess the biological significance of *miR-17-92* expression in ETS-dependent *Hras^G12V^* transformation, we generated *E1−E2−/Hras^G12V^* MEFs that stably express a *MSCV-puro-mir-17-92* retroviral vector. Forced expression of *pre*-*mir-17-92* in *E1−E2−/Hras^G12V^* cells led to increased expression of the 6 mature miRNAs (Figure 6A). A xenograft model revealed that all 8 subcutaneous sites injected with *E1−E2−/Hras^G12V^*/*MSCV-puro-miR-17-92* MEFs developed into tumors. None of the 8 sites injected with control *E1−E2−/MSCV-puro-miR-17-92* MEFs developed tumors (Figure 6B). There were no significant differences in tumor volumes between *E1−E2−/Hras^G12V^*/*MSCV-puro-mir-17-92* and *E1+E2+/Hras^G12V^* genotypes (Figure 6C, 6D).

## Conclusions

Transformation of immortalized mouse fibroblasts by *RAS* oncogenes provided a powerful assay for defining and characterizing the downstream signaling effectors of *RAS*, including ETS-family transcription factors [9], [19]–[22]. Previous work using dominant-negative approaches implicated ETS-family members as mediators of *RAS* transformation but were incapable of distinguishing which family members contributed to the transformed phenotype because multiple ETS family members with similar DNA binding properties are expressed in all cell lines and tissues [23], [24]. In the present work, we utilized null alleles of *Ets1* and *Ets2* to determine their function in *Hras^G12V^* transformation of immortalized MEFs. Expression of ETS1 and ETS2 was strongly induced in *Hras^G12V^* transformed cells compared to controls. Further, cells deficient for both factors were not transformed by *Hras^G12V^* as measured by both *in vitro* and *in vivo* assays. The effect of *Ets1* and *Ets2* are transformation-specific, as deletion of both factors had little effect on the growth of wild-type cells. These results definitively reveal a redundant function for *Ets1* and *Ets2* in *Hras^G12V^* transformation.

*c-Myc* acts as a central integrator of diverse signaling pathways that impact cell proliferation and cell growth [25]. Genetic evidence presented here demonstrated the requirement of *Ets1* and *Ets2* for expression of MYC in *Hras^G12V^* transformed cells. Further, ChIP experiments indicated that MYC is a direct target of the ETS-factors. ETS1 and ETS2 were recruited to a region of DNA that contains a regulatory element termed ME1a1, which is involved in chromatin organization required for the activity of the *c-Myc* P2 promoter [26]. Consistent with the function of this element, *RAS*-mediated phosphorylation of ETS1 and ETS2 promotes recruitment of CBP/p300 to target genes [27], indicating the ETS-factors may contribute to opening of chromatin and recruitment of elongation-competent RNA polymerase II complexes at the *c-Myc* P2 promoter [28], [29]. Importantly, expression of exogenous MYC in *Ets1–Ets2* null MEFs rescues transformation by *Hras^G12V^*, providing compelling evidence that *c-Myc* is a critical target for *Ets1*/*Ets2*-dependent transformation.

The essential role of *c-Myc* in *Ets1/Ets2* mediated *Hras^G12V^* transformation prompted the question of potential MYC target genes that could contribute to the transformed phenotype. One attractive target was the oncogenic *miR-17-92* cluster, a direct *c-Myc* target that is necessary for initiation and progression of *c-Myc*-induced B-cell lymphoma [14], [30]. Our results demonstrate that ETS-dependent *c-Myc* expression is required for efficient activation of the *miR-17-92* cluster. Additionally, MYC, ETS1, and ETS2 are recruited to *miR-17-92* cis-regulatory elements through conserved E-Box and ETS motifs and act together to superactivate expression of the cluster. The regulation of the *hTERT* gene is reported to require cooperation of *c-Myc* with *Ets-2*, indicating that collaboration between MYC and ETS1/ETS2 may be a more general mechanism for regulating genes involved in cell growth and cell survival [31]. Similar to *c-Myc*, *miR-17-92* overexpression rescued *Hras^G12V^* transformation in *Ets1/Ets2* deficient cells. These results suggest that *miR-17-92* is necessary for *Ets1–Ets2/Myc-*dependent transformation, but does not exclude the requirement for additional target genes that could be required for malignant transformation.

In summary, we have shown that *Ets1* and *Ets2* act in redundant fashion to elicit *Hras^G12V^*-mediated transformation of MEFs through the activation of *c-Myc* and *miR-17-92*. Previous work has implicated *Ets1*/*Ets2* and *c-Myc* collaboration in invasive breast cancer and in thyroid cancer in humans [32], [33]. Perhaps more relevant to the MEF model used here, amplification and overexpression of *c-Myc* or *N-myc* genes and *miR-17-92* have been reported in osteosarcoma and rhabmyosarcoma [34]–[37]. Whether our findings are relevant to these human cancers remains to be determined.

## Materials and Methods

### Animal Husbandry

*Ets1* knockout mice were provided by Dr. Muthusamy (The Ohio State University, Columbus, OH) [38]. Conditional *Ets2* loxP transgenic mice were generated previously described [39]. Eight to ten weeks old male nude mice were purchased from Taconic and housed and sacrificed in the BRT animal facility (Biomedical Research Tower) at the Ohio State University with accordance to the National Institute of Health regulations. The use of animals was approved by the Ohio State University Institutional Animal Care and Use Committee.

### Genotyping Primers and PCR Conditions

MEFs were genotyped by PCR method. The following primers that detect both Ets2 floxed and knockout alleles were used to confirm for *Ets2* deletion in MEFs: primer1 (TGAACTACTGTGTGTGACGAGGA), primer2 (GGAAGAAACGGGAAATCAAA), and primer3 (GGATTTTAGCCCAGAAACTTAGA). 2 µl of DNA was added to a total 20 µl PCR reaction and amplified employing the following PCR program: Cycle1∶95°C for 1 minute. Cycle2 was repeated 35x: 95°C (for 45 sec) followed by 58°C (for 45 sec) followed by 72°C (for 1 min). Cycle 3∶72°C (for 10 min). PCR products were run on 1.5% agarose gel.

### Cell Culture

Primary MEFs were generated from 13.5 days old embryos using standard methods and were spontaneously immortalized according to Todaro and Green protocol [40]. C-Myc^f/f^ established MEFs were a kind gift from Dr. Gustavo Leone’s laboratory at Ohio State University. Cells were grown in DMEM media supplemented with 10% FBS and Penicillin and Streptomycin antibiotics.

### Retroviral Infection of MEFs

Phoenix retrovirus packaging cells were transfected by calcium phosphate method with 8 µg of DNA per 60 mm dish of the following retroviral vectors: pBabe-Hygromycin-*H-Rasv12*, pBabe-Hygromycin-empty-vector, pBabe-Puromycin-*Cre*, pBabe-Puromycin-empty-vector, MSCV-Puromycin-*miR-17-92*, MSCV-Puromycin-empty vector, MSCV-GFP-*c-Myc* (kind gift from Dr. Leone laboratory) and MSCV-GFP empty vector. High titers of retrovirus supernatant supplemented with 4 µg/µl of Sequabrene (Sigma) to increase retrovirus uptake by cells were used to infect MEFs twice at 24 and 48 hrs time points. Infected cells were treated with 4 µg/µl of Puromycin for 3 days and or 200 µg/µl of Hygromycin for 5 days to select for stably mixed specific cellular genotypes. MEFs infected with the retroviral GFP vectors were sorted for GFP selection by the FACS/Aria machine.

### Anchorage dependent and independent cellular growth assays

For anchorage dependent cellular growth, cells of different genotypes were seeded in 4 replicate wells at a density of 1×10^4^ cells per well in 6-well plates. At days 2,4 and 6 after seeding, cells were washed with 1x PBS, trypsinized, mixed with 0.4% Trypan blue solution (Sigma Aldrich) and then counted using the Reichert Bright-Line Hemacytometer (Hausser Scientific) by Trypan blue exclusion principle. For anchorage independent cellular growth, cells of different genotypes were grown in 4 replicate wells in two layers 6-well plates: The lower layer consisted of 0.6% (w/v) soft agar and the upper layer contained 1×10^4^ cells per well mixed in 0.3% (w/v) soft agar. Both soft agar layers consisted of 20% FBS. After 14 to 21 days, grown cellular colonies were scored.

### Tumorigenic assay

Animal studies were performed with 8–10 weeks old male athymic nude mice. Cells were harvested, counted and suspended in PBS at a concentration of 1×10^6^ cells per injection site. 100 µl of cells were injected subcutaneously into the right and left shoulders and hips (four injections per mouse), or two injections per mouse (right and left hips). Tumors were harvested after 3 to 4 weeks before reaching 1 cm in length.

### Quantitative real-time PCR

RNA was extracted from MEFs by Trizol (Invitrogen) according to the manufacturer instructions. The cDNA was prepared as described previously [41]. Most of the RNA primers used for this study were designed using Roche Universal Probe Library System. To avoid non specificity in mature RNA detection, intron-spanning primers were designed. The following RNA primers were used: mouse *c-Myc* primers: forward primer (CCTAGTGCTGCATGAGGAGAC), reverse primer (CCTCATCTTCTTGCTCTTCTTCA). Mouse pre-*miR-17-92* primers: forwards primer (TCTGACAATGTGGAGGACAGA), reverse primer (CCTTTAGAGGAAAGCCTCACATT). Mouse *RpL4* primers: forwards primer (AGCAGCCGGGTAGAGAGG), reverse primer (ATGACTCTCCCTTTTCGGAGT). RNA expression analysis of the different sets of genes had their threshold adjusted in accordance to ribosomal *RpL4* gene expression. Relative quantification was calculated using the 2-ΔΔCT relative quantification method [41].

### Western Blot Analysis

MEFs were lysed in RIPA buffer (50 mM Tris-HCl (pH7.4), 1% NP-40, 0.25% Na-Deoxycholate, 150 mM NaCl, and 1 mM EDTA) for 30 minutes. The following inhibitors were added to RIPA buffer (1 mM PMSF, 1 µg/ml Aprotinin, 1 µg/ml Leupeptin, 1 µg/ml Antipain, 1 mM Na_3_VO_4_). The lysate was centrifuged at 14,000 g at 4°C for 30 minutes. Aliquots were made from the supernatant and stored at −70°C. Protein concentration was measured by the Bradford assay. 20 µg of proteins were run on 10 to 12% SDS-Polyacrylamide gels. Nitrocellulose membranes were used for protein transfer. Membranes were blocked with (5% non-fat dry milk in 0.05%TBST) for 1 hr at room temperature, then incubated with the following primary antibodies overnight at 4°C: MYC (sc-40, Santa Cruz), α-Tubulin antibody (Sigma), β-Actin (sc-47778, Santa Cruz), Pan-Ras (OP-40, CalBiochem), ETS1 and ETS2 antibodies were prepared in our laboratory as described in the previous section of materials and methods. Nitrocellulose membranes were then incubated with a horse radish peroxidase-conjugated secondary antibody (either rabbit or mouse) for 1 hr and developed using the ECL chemiluminescence system (Thermo Scientific).

### Chromatin Immunoprecipitation Assay (ChIP)

MEFs were seeded at a density of 1×10^6^ in 100 mm dish and protein/DNA cross-linking was performed using 270 µl of formaldehyde at 1% final concentration at room temperature for 10 min. The rest of the assay was performed as described [39]. Briefly the DNA-protein complexes were immunoprecipitated with 2 µg of antibodies overnight at 4°C. After elution, the eluted solution was heated at 65°C overnight then DNA was precipitated with 70% ethanol and kept at −20°C overnight. DNA was recovered and purified using the Qiagen PCR purification kit according to the manufacturer’s instructions. SYBR Green primers were used for *miR-17-92* promoter detection: forward primer (GGGCTCGGAAAGTG), reverse primer (ACTCACCCACTCAG). For *c-Myc* promoter detection, probe 16 from the Universal Primer library (Roche Diagnostics, Indianapolis, IN) was used with forward primer (GTCCGACTCGCCTCACTC) and reverse primer (CCTCCCCTCCCTTCTTTTT **)**. Rabbit IgG (Millipore) was used as control and the antibodies were the same as for Western analysis. The threshold value for the promoter being studied was normalized to that of input values and represented as relative enrichment relative to IgG values.

### MicroRNA gene expression analysis

Total RNA was extracted from cells using Trizol reagent (Invitrogen). The cDNA was subsequently synthesized from 5 ug RNA by oligo dT primers and superscript II (Invitrogen). Quantitative analysis of mature microRNA expression was performed by real-time PCR using Taqman microRNAs assays (Applied Biosystems). For normalization of expression levels U6 snRNA and sno RNA 202 (Applied Biosystems) were used. Real-time quantitative RT-PCR experiments were performed in the ABI Prism 7700 System (Applied Biosystems). Real time PCR was done on all of the following mature microRNAs of the *miR-17-92* Cluster: *miR-17*, *miR-18*, *miR-19a*, *miR-19b*, *miR-20* and *miR-92*. Relative quantification was calculated using the ^2−ΔΔCT^ relative quantification method.

### Transient transfection

The following DNA vectors were transiently transfected into c-Myc^−/−^ MEFs using lipofectamine-2000 protocol according to Invitrogen manual instructions: FNEts1, FNEts2, FNpcDNA3, pBabe-Hygromycin-empty and pBabe-Hygromycin-c-Myc. *C-Myc^−/−^* MEFs seeded in 4 replicate 60 mm-dishes for each experimental condition at a density of 3.5×10^5^ cells per dish, were transfected with 4 µg of DNA vector mixed with 15 µl of Lipofectamine-2000 in plain DMEM. The transfection cocktail was kept for 6 hrs then replaced with 10%FBS media for 36 hrs. Afterwards, cells were lysed with trizol and kept at −70°C until further RNA processing.

### Statistics

Statistical analysis was done using the standard deviation formula and the student *t-test* to determine the statistical significance between the control and experimental genotypes.

## Supporting Information

Figure S1 A) PCR genotyping results for the different MEFs after infection with Cre recombinase retroviral vector showing *Ets2* flox and *Ets2* knockout bands. The last two PCR bands represent two positive control samples with either *Ets2* flox or *Ets2* knockout band. B) Growth of *E1+ E2+* and *E1− E2−* MEFs was assessed by trypan blue exclusion at day 2 and day 4 post-seeding. C) Pie chart representing cell cycle distribution after flow cytometry analysis of Propidium Iodide stained *E1+E2+* and *E1−E2−* cells. D) Graph representing growth at day 6 post-cellular seeding by trypan blue exclusion of indicated cellular genotypes. E) Graph representing percentage of BrdU stained cells in the indicated MEFs genotypes. Asterisk indicates P<0.05.
(PDF)

Figure S2 A) Bar graphs showing number of colonies growing in soft agar assays (see Materials & Methods) for MEFs of the indicated genotypes. B) Graph representing tumor volumes of the indicated genetic groups. B) PCR genotyping result for the single tumor that grew from the *E1−E2−/H-Rasv12* injected cells (lane M). The other two lanes represent two positive control samples containing either *Ets2* flox or *Ets2* knockout band.
(PDF)
