# Supplementary figures and images for: MicroRNA 17-92 Cluster Mediates ETS1 and ETS2-Dependent RAS-Oncogenic Transformation
Source: PLoS One. 2014 Jun 26;9(6):e100693. doi: 10.1371/journal.pone.0100693 (PMC4072627; doi:10.1371/journal.pone.0100693)

## Supplementary Figure S1

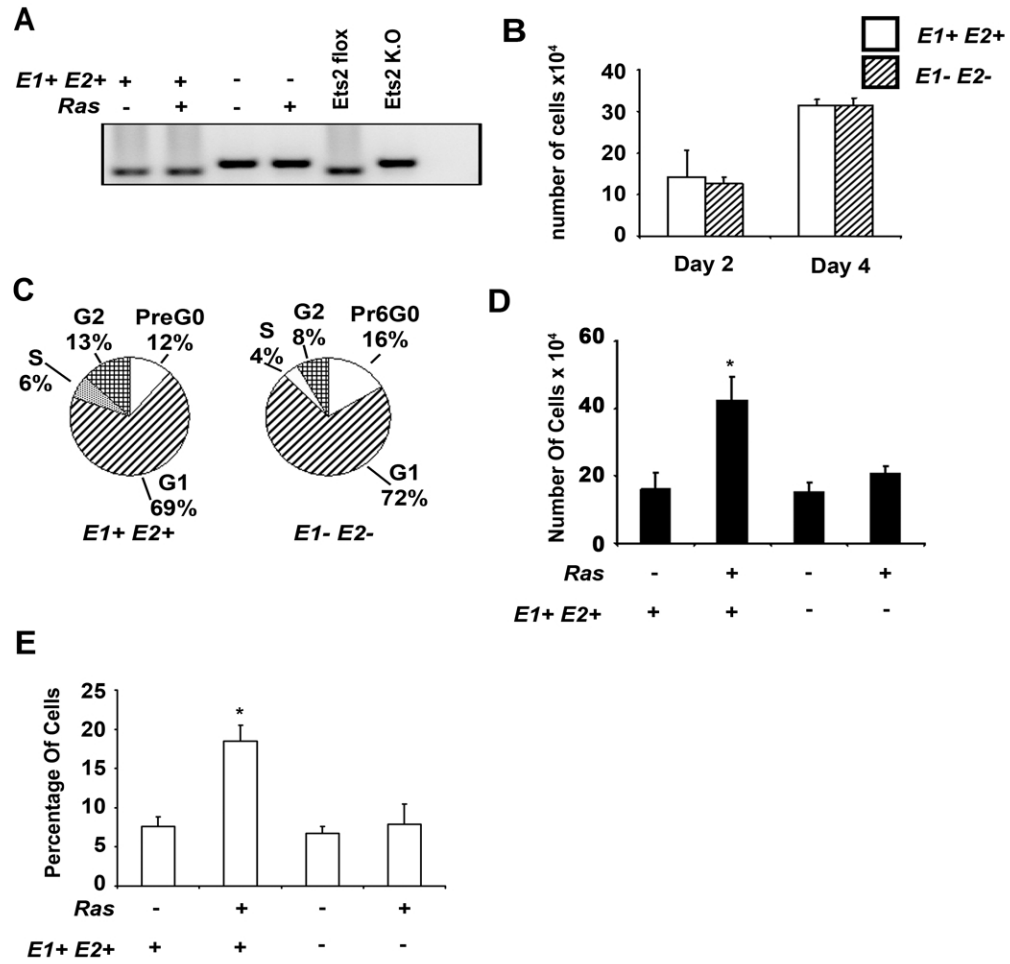

Supplement: Figure S1 — A) PCR genotyping results for the different MEFs after infection with Cre recombinase retroviral vector showing Ets2 flox and Ets2 knockout bands. The last two PCR bands represent two positive control samples with either Ets2 flox or Ets2 knockout band. B) Growth of E1+ E2+ and E1− E2− MEFs was assessed by trypan blue exclusion at day 2 and day 4 post-seeding. C) Pie chart representing cell cycle distribution after flow cytometry analysis of Propidium Iodide stained E1+E2+ and E1−E2− cells. D) Graph representing growth at day 6 post-cellular seeding by trypan blue exclusion of indicated cellular genotypes. E) Graph representing percentage of BrdU stained cells in the indicated MEFs genotypes. Asterisk indicates P<0.05. (PDF) [file pone.0100693.s001.pdf]

Supplementary Figure S2

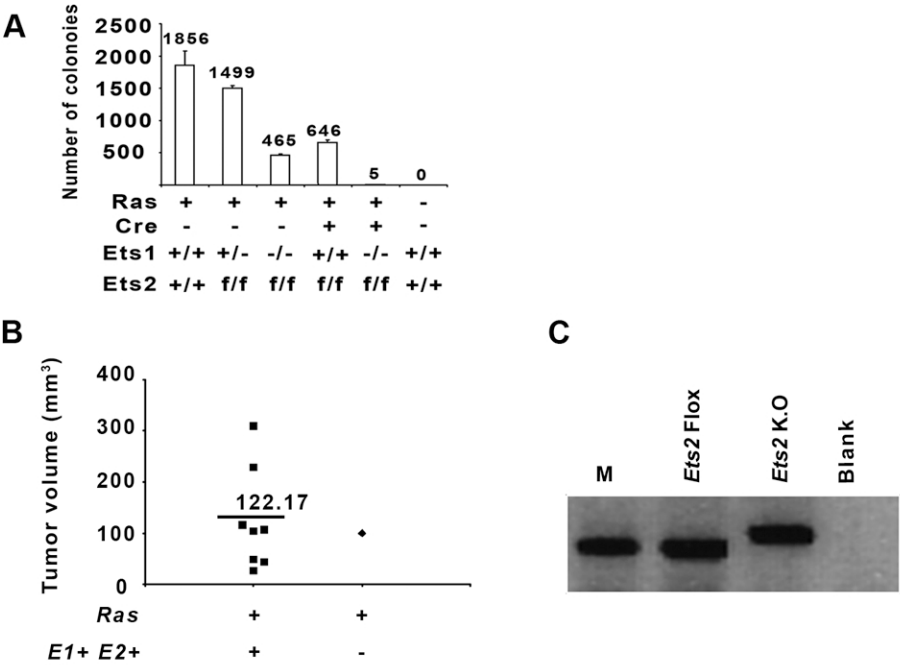

Supplement: Figure S2 — A) Bar graphs showing number of colonies growing in soft agar assays (see Materials & Methods) for MEFs of the indicated genotypes. B) Graph representing tumor volumes of the indicated genetic groups. B) PCR genotyping result for the single tumor that grew from the E1−E2−/H-Rasv12 injected cells (lane M). The other two lanes represent two positive control samples containing either Ets2 flox or Ets2 knockout band. (PDF) [file pone.0100693.s002.pdf]
